# Development of a Disposable Single-Nozzle Printhead for 3D Bioprinting of Continuous Multi-Material Constructs

**DOI:** 10.3390/mi11050459

**Published:** 2020-04-28

**Authors:** Tiffany Cameron, Emad Naseri, Ben MacCallum, Ali Ahmadi

**Affiliations:** Faculty of Sustainable Design Engineering, University of Prince Edward Island, Charlottetown, PE C1A 4P3, Canada

**Keywords:** multi-material printing, 3D bioprinting, continuous multi-material printing, single-nozzle printing

## Abstract

Fabricating multi-cell constructs in complex geometries is essential in the field of tissue engineering, and three-dimensional (3D) bioprinting is widely used for this purpose. To enhance the biological and mechanical integrity of the printed constructs, continuous single-nozzle printing is required. In this paper, a novel single-nozzle printhead for 3D bioprinting of multi-material constructs was developed and characterized. The single-nozzle multi-material bioprinting was achieved via a disposable, inexpensive, multi-fuse IV extension set; the printhead can print up to four different biomaterials. The transition distance of the developed printhead was characterized over a range of pressures and needle inner diameters. Finally, the transition distance was decreased by applying a silicon coating to the inner channels of the printhead.

## 1. Introduction

Three-dimensional (3D) bioprinting aims to fabricate artificial tissues [1,2,3,4,5]. Artificial tissues consist of three primary elements: (a) an extracellular matrix (ECM), (b) a vascular system, and (c) multiple cell types [6,7,8,9]. 3D bioprinting techniques are used to precisely control the spatial and temporal position of cells within the ECM to fabricate the artificial tissues [3]. To achieve the high level of complexity needed for tissue fabrication, 3D bioprinting techniques including inkjet [10,11,12,13] laser-assisted [14], and micro-extrusion [15,16,17,18,19] have been explored. Micro-extrusion systems, in particular, have been widely used to facilitate multiple material printing at low temperatures due to their high level of compatibility with a wide range of cells.

One of the most common techniques for micro-extrusion of multi-materials is the use of multiple nozzles, each dispensing a filament of a single material or cell type. These multi-nozzle 3D bioprinters have the inherent disadvantage of increasing the printing time when switching between materials, as well as requiring precise calibration for all the printheads before each print [19,20]. Furthermore, the multi-nozzle bioprinting leads to a discontinuity in the filament which compromises the mechanical integrity of the printed constructs [19]. The ability to print multiple materials through a single nozzle aims to overcome these limitations.

Continuous single-nozzle multi-material (SNMM) micro-extrusion bioprinting is achieved by sequentially dispensing different materials out of a single nozzle. Different techniques have been used for fabricating SNMM printheads. Soft lithography techniques have been used to create microfluidic chips which are used as SNMM printheads to fabricate vascularized tissues, textured composites, and multi-material spheroids [21,22,23,24,25]. Custom-engineering techniques have also been used to fabricate SNMM printheads. Hardin et al. developed a two-material single-nozzle printhead using CNC-milling with solvent welding [26]. Lui et al. developed a seven-material printhead using heat to pull glass capillaries into a single outlet [27]. Nadernezhad et al. developed a printing system that was able to sequentially aspirate different materials to form heterogeneous hydrogel scaffolds [28]. These microfluidic chips and custom-engineering solutions require costly equipment and expertise for fabrication which limits the application of SNMM printheads. Moreover, cross-contamination has been reported to be a major issue for many of these SNMM bioprinting technologies. These constraints highlight the need for an affordable and disposable SNMM bioprinting technology.

In this study, a readily available, disposable, and low-cost SNMM printhead was developed. The transition distance between the two materials was characterized using MATLAB^®^ image analysis. The effects of nozzle diameter and extrusion pressure on the transition distance were characterized. Finally, it was shown that the transition distance of the dispensed bioinks through the SNMM printhead can be reduced using a silicone coating for the inner channels of the printhead.

## 2. Materials and Methods

### 2.1. Printhead Set-up

**Figure 1:**
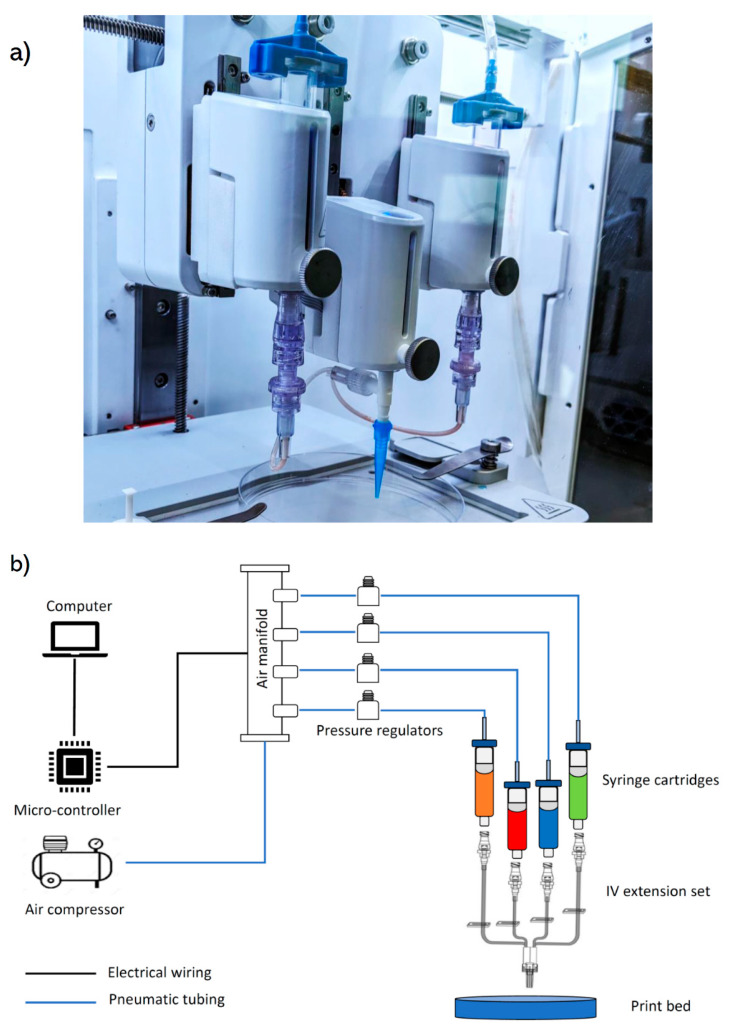
(**a**) Assembly of the SNMM printhead on a commercial BioX 3D bioprinter, (**b**) schematic diagram of the SNMM printing set-up.

As shown in Figure 1a, the printhead was assembled onto a commercial 3D bioprinter (BioX, Cellink, Gothenburg, Sweden). A pneumatically driven printing system was assembled using an external pressure source in conjunction with an air manifold (5203K919, McMaster-Carr, Elmhurst, IL, USA) and air regulators (8083T2, McMaster-Carr, Elmhurst, IL, USA). Pneumatic pressure lines were connected to 5 mL syringe cartridges (7012096, Nordson EFD, Westlake, OH, USA) via adaptive tubing connectors (7012062, Nordson EFD, Westlake, OH, USA). An Arduino-compatible micro-controller (Kuman, Amazon, Washington, WA, USA) was used to control pressure and switching delay in the air manifold. To prevent back flow, the syringe cartridges were connected to an intravenous (IV) extension set (179-MC33634, ICU Medical, San Clemente, CA, USA) via luer-lock connections. Conical needles with inner diameter of 200, 250, 330, and 430 μm (6699A8, 6699A7, 6699A6, 6699A5, McMaster-Carr, Elmhurst, IL, USA) were connected to the outlet of the IV extension set via luer-lock connections (Figure 1b).

### 2.2. Material Selection

A starch-based polymer, which is biodegradable and biocompatible, was used as the test material [29]. Water was used as a solvent, and glycerol monostearate (4.55% w/v) (Alfa Aesar™, Fisher Scientific, Hampton, VA, USA) was used as a surfactant. Water was heated to 66 °C and glycerol monostearate was added and stirred at 300 rpm for 2 min. Furthermore, potato starch (12.12% w/v) (Bob’s Red Mill Natural Foods, Milwaukee, OR, USA) was added and stirred manually for 1 min followed by mechanical stirring for 6 min at 170 rpm off heat. Finally, the solution was dyed with liquid food coloring (AmeriColor, Placentia, CA, USA).

### 2.3. Experimental Process

Two 5 mL syringe cartridges containing starch-based bioink in different colors, red and blue, were used for the experimental prints. The printed designs were created using a computer-aided design (CAD) software (SolidWorks, Waltham, MA, USA), and G-codes were generated with a slicer software (Slic3r, Repetier-Host, Willich, Germany). The bioprinter was calibrated in *x*, *y*, and *z* directions, and the printing speed was determined before each print. An external air pressure source was used, and the pressure was further regulated by the micro-controller. A standard switching delay of eight seconds was specified within the Arduino code for the print tests (Arduino 3, Version 1.8.9).

### 2.4. Image Analysis

**Figure 2:**
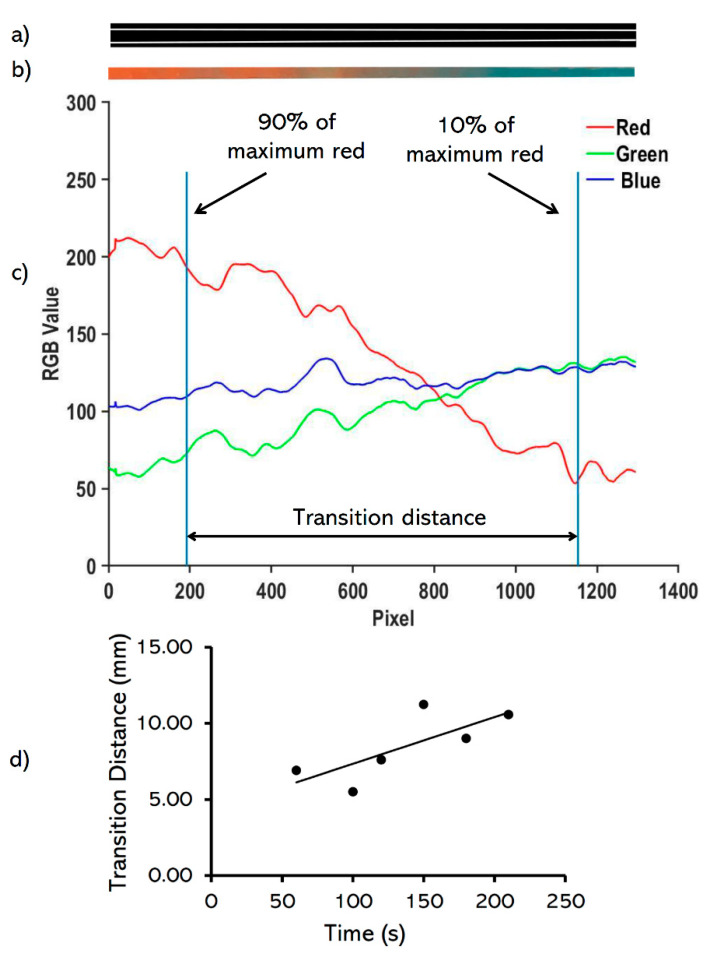
Image analysis of the printed red and blue bioinks. (**a**) converting of the cropped image to black and white to detect the filament edges, (**b**) cropping the transition area of the printed pattern, (**c**) determining the transition distance within 90% of the maximum red value to 10% of the maximum red value while switching from red to blue (**d**) identifying the rate of change in the transition distance (*D_tr_* = 0.03060 × *t*), where *D_tr_* is the transition distance (in mm) and *t* is time (in·s).

A MATLAB-based image processing script (MathWorks, Natick, MA, USA) was created to determine the transition distance between the red and blue bioinks. The images of the transition zone within printed filaments were converted to black and white, and Canny edge detection method (threshold = 0.3) was applied (Figure 2a). The RGB values of the pixels within the detected edges were averaged and a moving average of 20 pixels was used (Figure 2b). The transition distance was defined as the length of filament required for transition from 90% of the maximum red value to 10% of the maximum red value while switching from red to blue, as shown in Figure 2c. Finally, the transition distance in pixels was converted to millimeters. All measurements were calculated with an uncertainty of 0.02 mm. 

### 2.5. Diffusion Rate Characterization

To determine the transition distance independent of bioink physical properties, the effect of diffusion of dye within the printed filament after printing was characterized, and the transition distance values were subsequently modified. To this aim, the transition distance of a printed filament via a 430 μm nozzle in 45 psi was measured over time after printing (Figure 2d), and the graph linear trendline was used to modify transition distances for the experiments. The diffusion-related transition distances were subtracted from the measured transition distances to determine the real transition distance values.

### 2.6. Hydrophobic Coating of the Printhead

The inside of the printhead (IV extension set and conical needle) was coated with a solution of silicone (SurfaSil Siliconizing Fluid, ThermoFisher Scientific, Waltham, MA, USA) in acetone (Sigma-Aldrich, St. Louis, MO, USA) (95% v/v) and the printhead was left to dry overnight. The chemical composition of the SurfaSil is known as tetrasiloxane, 1,7-dichloro-1,1,3,3,5,5,7,7-octamethyl and it is often used to render hydrophilic surfaces hydrophobic [30,31,32].

## 3. Results and Discussion

### 3.1. Printing Integrity

**Figure 3:**
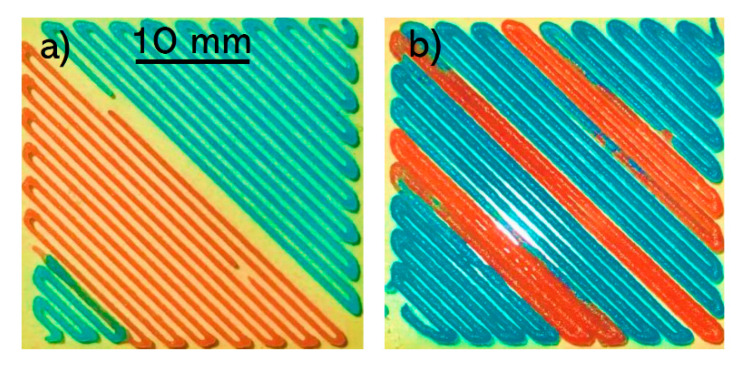
Comparison of a 30 mm × 30 mm square printed by (**a**) the commercially available multi-head bioprinter and (**b**) the developed SNMM printhead.

To investigate the continuous multi-material printing capability of the developed printing system in comparison to a commercially available multi-head printing system, a 30 × 30 mm pattern was printed (Figure 3). The printed construct via the commercially available system demonstrated a discontinuity of printing filament while changing the bioink from red to blue (Figure 3a). This discontinuity was not observed when using the developed SNMM printhead as shown in Figure 3b. 

### 3.2. Printing Different Geometries 

**Figure 4:**
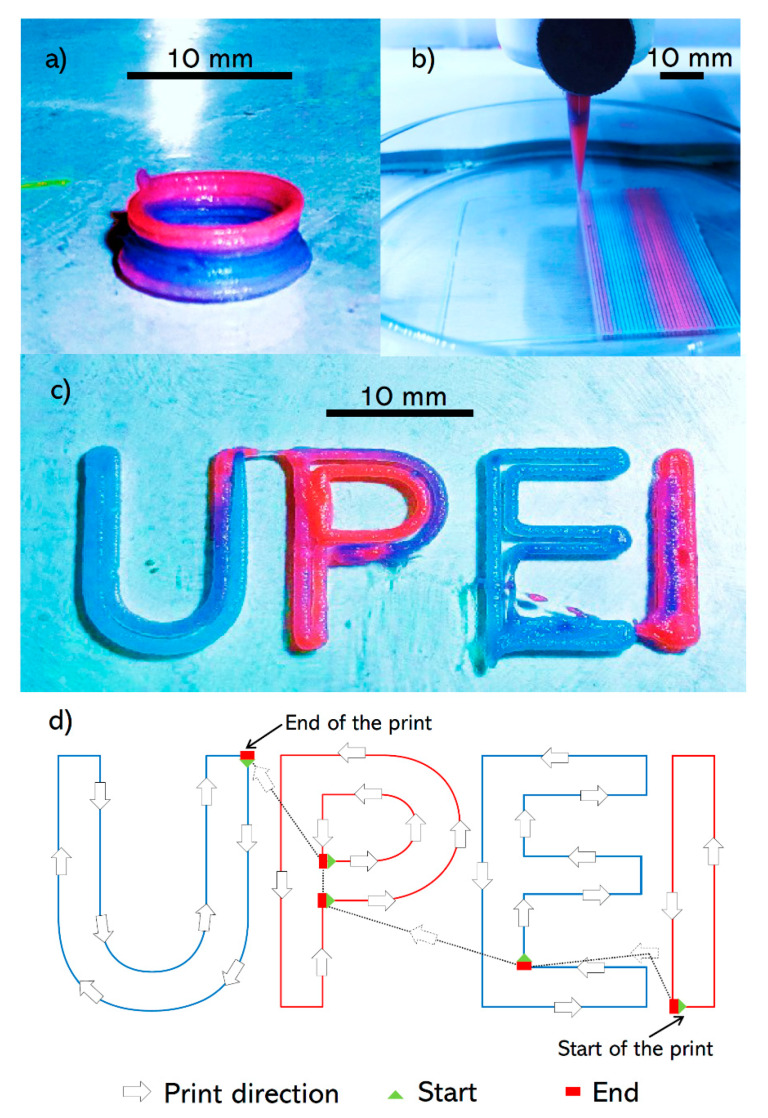
Different geometries printed with the SNMM printhead: (**a**) cylinder (10 mm diameter, 5 mm height) using a 430 µm nozzle, (**b**) square (50 mm × 50 mm) using a 200 µm nozzle and (**c**) UPEI letters using a 430 µm nozzle. (**d**) Printhead path and transition of bioinks in UPEI letters.

The developed SNMM printhead was used to print different geometries including a cylinder, a square, and UPEI letters (Figure 4). The constructs were printed continuously and demonstrated a seamless transition between the two bioinks. The color transition in the cylinder occurred during one layer (Figure 4a). The transition distance in the printed square is noticeably larger on the right side of the image than the other side (Figure 4b). This highlights the effect of diffusion of the dye within the filament after printing which explains the lower apparent transition distance in the freshly-printed filaments compared to the rest of the filaments. In UPEI letters, the print started from I and finished at U (Figure 4c). The transitions from red to blue occurred mainly within I and P letters. Although other travel moves between the letters may also be viable, they may lead to printing of additional material or going over the printed filaments and therefore diminishing the printing quality. 

### 3.3. Characterizing the Transition Distance

To characterize the printhead for high-resolution printing, a pressure range of 25–55 psi and nozzle outlet diameters of 250, 330, and 430 μm were used while printing. It should be noted that with the 200 μm nozzle size, the printing speed was too high and not controllable at high pressures, and therefore, no data is presented for this nozzle size.

**Figure 5:**
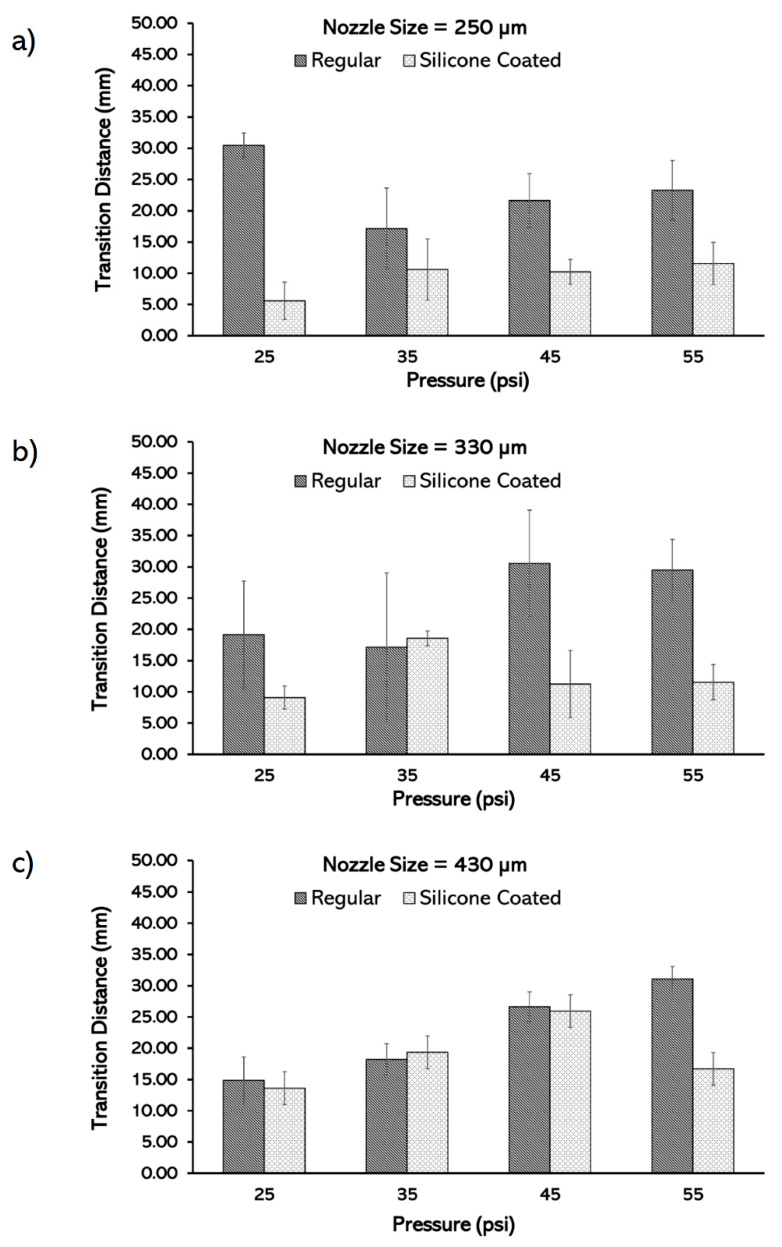
Characterization of the transition distance based on pressure in regular and silicone-coated printheads in different nozzle sizes: (**a**) 250 µm, (**b**) 330 µm, and (**c**) 430 µm.

Overall, for all the nozzle sizes in regular printheads (with no coating), with a few exceptions, increasing the pressure increases the transition distance (Figure 5). The smallest transition distance achieved was 14.87 mm for nozzle diameter of 430 μm and pressure of 25 psi. For the regular printheads at lower pressures, the transition distance decreases as the nozzle diameter increases, whereas no strong dependency on the nozzle size is observed at higher pressures. Therefore, when regular printheads are used, using larger nozzles and smaller pressures are recommended to achieve smaller transition distances. 

The use of silicone coating decreased the transition distance as well as the variation in transition distance. In larger nozzles, the coating of the printhead is less effective in reducing the transition distance due to the smaller surface area to volume ratio. The silicone coating reduced the smallest transition distance for the 430, 330, and 250 μm regular printheads from 14.87, 17.12, 17.16 to 13.63, 9.10, 5.60, respectively (Figure 5). The silicone coating effect on the transition distance is highlighted in smaller nozzles, with the greatest effect shown in the 250 μm nozzle at 25 psi. In addition, compared to the coated printheads, the transition distance in the regular printheads are more sensitive to the pressure changes. Moreover, at a given pressure, the transition distance decreases as the nozzle diameter decreases in coated printheads, while in the regular printheads, the transition distance increases (Figure 5). It is recommended to use a silicone coating as well as the smallest nozzle size at low pressures to minimize the transition distances associated with the SNMM. 

## 4. Conclusions

In this article, a single-nozzle readily available printhead was developed to be used for creating structurally stable, high-resolution, and custom bioprinted constructs. It is shown that by coating of the nozzle using a hydrophobic layer the transition distance decreases. It is recommended to use a silicone coating as well as the smallest nozzle size at low pressures to minimize the transition distances associated with the SNMM. The use of this printhead facilitates the research of multi-material tissue structures by providing an inexpensive printhead which ensures faster tissue engineering prototyping; it is readily available and requires low-maintenance. The maximum shear rate within the tubing and nozzle is calculated to be ~4.2 s^−1^ and 25.0 s^−1^, respectively. These shear rates are small, and it has been shown in the literature that much higher shear rates are needed to negatively affect the cell viability for the range of bioink viscosities used in this study [33]. In addition, this printhead is a suitable tool to mimic gradient and interconnected tissue structures which is essential for understanding the cell-to-cell interactions within the human body.
